# Does Indocyanine Green Utilization during Esophagectomy Prevent Anastomotic Leaks? Systematic Review and Meta-Analysis

**DOI:** 10.3390/jcm13164899

**Published:** 2024-08-20

**Authors:** Andrea Sozzi, Davide Bona, Marcus Yeow, Tamer A. A. M. Habeeb, Gianluca Bonitta, Michele Manara, Giuseppe Sangiorgio, Antonio Biondi, Luigi Bonavina, Alberto Aiolfi

**Affiliations:** 1 I.R.C.C.S. Ospedale Galeazzi—Sant’Ambrogio, Department of Biomedical Science for Health, Division of General Surgery, University of Milan, 20122 Milano, Italy; sozzi94@hotmail.it (A.S.); davide.bona@unimi.it (D.B.); bbonit@icloud.com (G.B.); michele.mnra@gmail.com (M.M.); 2 Department of Surgery, National University Hospital, National University Health System, 1E, Kent Ridge Road, NUHS Tower Block, Level 8, Singapore 119228, Singapore; m.yeow123@gmail.com; 3 Department of General Surgery, Faculty of Medicine, Zagazig University, Zagazig 7120001, Egypt; tameralnaimy@hotmail.com; 4 Department of General Surgery and Medical Surgical Specialties, Surgical Division, G. Rodolico Hospital, University of Catania, 95131 Catania, Italy; giuseppe.sangiorgio@phd.unict.it (G.S.); abiondi@unict.it (A.B.); 5 I.R.C.C.S. Policlinico San Donato, Department of Biomedical Sciences for Health, Division of General and Foregut Surgery, University of Milan, 20097 Milan, Italy; luigi.bonavina@unimi.it

**Keywords:** esophagectomy, anastomotic leak, gastric conduit perfusion, indocyanine green, fluorescence

## Abstract

**Background:** Indocyanine Green (ICG) is a promising technique for the assessment of gastric conduit and anastomosis perfusion during esophagectomy. ICG integration may be helpful in minimizing the risk of anastomotic leak (AL). Literature evidence is sparse, while the real effect of ICG assessment on AL minimization remains unsolved. The aim of this systematic review and meta-analysis was to compare short-term outcomes between ICG-guided and non-ICG-guided (nICG) esophagogastric anastomosis during esophagectomy for cancer. **Materials and Methods:** PubMed, MEDLINE, Scopus, Web of Science, Cochrane Central Library, and ClinicalTrials.gov were queried up to 25 April 2024. Studies that reported short-term outcomes for ICG versus non-ICG-guided (nICG) anastomosis in patients undergoing esophagectomy were considered. Primary outcome was AL. Risk ratio (RR) and standardized mean difference (SMD) were utilized as effect size measures, whereas to assess relative inference we used 95% confidence intervals (95% CI). **Results:** Overall, 1399 patients (11 observational studies) were included. Overall, 576 (41.2%) underwent ICG gastric conduit assessment. The patients’ ages ranged from 22 to 91 years, with 73% being male. The cumulative incidence of AL was 10.4% for ICG and 15.4% for nICG. Compared to nICG, ICG utilization was related to a reduced risk for postoperative AL (RR 0.48; 95% CI 0.23–0.99; *p* = 0.05). No differences were found in terms of pulmonary complications (RR 0.83), operative time (SMD −0.47), hospital length of stay (SMD −0.16), or 90-day mortality (RR 1.70). **Conclusions:** Our study seems to indicate a potential impact of ICG in reducing post-esophagectomy AL. However, because of limitations in the design of the included studies, allocation/reporting bias, variable definitions of AL, and heterogeneity in ICG use, caution is required to avoid potential overestimation of the ICG effect.

## 1. Introduction

Esophageal cancer is the eighth leading cause of cancer and the sixth cause of oncological death worldwide [1]. The gold standard treatment is esophagectomy, lymphadenectomy, a reconstruction of the digestive tract through stomach tubulization, and esophagogastric anastomosis, either thoracic or cervical [2,3]. The procedure is technically complex and burdened by frequent complications. The most feared surgical complication is anastomotic leak (AL), which occurs in approximately 10% of cases [4]. AL has been shown to be associated with an increased risk of reintervention, prolonged hospitalization, the delayed initiation of oral feeding, increased 90-day mortality, and worsened long-term oncological outcomes [5,6,7,8,9].

Various risk factors have been linked to a higher risk of postoperative anastomotic leak. Among these, inadequate blood supply to the tip of the gastric conduit (GC) has been identified as a major contributing factor [4,10,11,12,13,14,15,16]. Evaluating the perfusion of the GC can be carried out subjectively by assessing its colour, temperature, the bleeding from the incision edges, and the pulsation of marginal vessels supplied by the right gastroepiploic artery. However, these clinical estimations have a low accuracy and predictive value for AL [17]. Objective measurements with laser Doppler flowmetry and tissue pulse oximetry have been attempted but concerns about their reproducibility and the lack of correlation between perfusion data and AL have limited their utilization [10]. In contrast, indocyanine green (ICG) fluorescence angiography has become widely accepted due to its easy reproducibility and low cost [10,11]. Previous meta-analyses have been published with diverging results and methodological flaws because of the inclusion of single-arm studies [18,19,20].

The purpose of this study was to perform a systematic literature review and meta-analysis comparing short-term outcomes between ICG and non-ICG-guided (nICG) esophagogastric anastomosis during esophagectomy for cancer.

## 2. Materials and Methods

This study was conducted following the Preferred Reporting Items for Systematic Reviews and Meta-Analyses (PRISMA) statement and Meta-analyses Of Observational Studies in Epidemiology (MOOSE) guidelines [21,22]. Institutional Review Board approval was not required. PubMed, MEDLINE, Scopus, Web of Science, Cochrane Central Library, and ClinicalTrials.gov were queried. The complete search strategy is described in Appendix A. The last date of the search was 25 April 2024. A combination of the following MeSH (Medical Subject Headings) terms was utilized for the literature search: (“esophagectomy” (tiab) OR “oesophagectomy” (tiab)) AND (“indo-cyanine green” (tiab) OR “ICG” (tiab)) AND (“outcomes” (tiab) OR “complication” (tiab)) AND (“leak” (tiab) OR “leakage” (tiab)). AS and AA reviewed the title of each study and extracted relevant abstracts. The search was further enhanced by examining the references of each article. The study protocol has been registered with the international prospective register of systematic reviews (PROSPERO registration number: CRD42024542776) [23].

### 2.1. Eligibility Criteria

The criteria for inclusion were (a) cohort studies comparing outcomes of ICG versus nICG in patients undergoing elective esophagectomy for esophageal cancer (b) written in English; (c) in cases where multiple studies were based on the same dataset, the study with the longest follow-up period or the largest sample size was selected, and (d) for duplicate studies, only the study with the most complete dataset was included for quantitative analysis. The exclusion criteria were (a) non-English articles, (b) single-arm studies reporting data exclusively for ICG patients, (c) studies with a lack of clear outcome differentiation between ICG and nICG, and (d) studies with less than 10 patients in the treatment arm.

### 2.2. Data Extraction

The following variables were collected: the authors, year of publication, country, study design, the number of patients, age, sex, body mass index (BMI), American Society of Anaesthesiologists (ASA) physical status, comorbid conditions, surgical indications, tumour characteristics, histological type, tumour location, cancer stage, the use of neoadjuvant chemoradiation therapy, and postoperative outcomes. Three investigators (AA, AS, MM) independently gathered and analyzed the data, which were then compared at the conclusion of the review. Discrepancies were resolved through discussion.

### 2.3. Outcomes

Primary outcome was AL. Secondary outcomes were postoperative pulmonary complications (PCs), operative time (OT) in minutes, hospital length of stay (HLOS) in days, and 90-day mortality. AL was defined as radiographic evidence of contrast extravasation upon postoperative swallow study and/or computed tomography, or the endoscopic visualization of anastomotic dehiscence/fistula, or a surgical drain output consistent with saliva. Change in management after perfusion assessment was defined as need for additional GC tip resection or a substantial change of anastomosis location.

### 2.4. Quality Assessment

Three authors (AA, AS, MM) performed separate evaluations of the methodological quality of each study. The ROBINS-I tool was used, taking into account confounding bias, selection bias, classification bias, intervention bias, missing data bias, outcome measurement bias, and the bias in study reporting [24]. Each domain was rated as “yes”, “probably yes”, “probably no”, or “no”, leading to overall risk judgments categorized as a low, moderate, serious, or critical bias risk. The quality of the overall evidence from the studies was evaluated using the Grading of Recommendations, Assessment, Development, and Evaluation (GRADE) tool (https://www.gradepro.org; accessed on 20 May 2024) [25].

### 2.5. Statistical Analysis

The results of the systematic review were defined using a random-effects frequentist meta-analysis to compute pooled risk ratios (RRs) and standardized mean differences (SMDs). An inverse-variance method and the DerSimonian–Laird estimator (τ^2^) were applied [26]. Heterogeneity among studies was gauged using the I^2^ index and Cochran’s Q test [27]. Statistical heterogeneity was classified as low (<25%), moderate (25–75%), or high (>75%) [28]. A Wald-type 95% confidence interval (CI) was computed. Otherwise, the 95% CI for the I^2^ index was determined as per Higgins and Thompson [29]. The prediction interval was calculated according to Borestein [27]. One-leave-out sensitivity analysis was conducted by excluding one study at a time and reanalyzing the data to ensure the robustness of results. The Trim and Fill funnel plot and Egger’s test were used to estimate publication bias. A two-sided *p*-value was deemed statistically significant (*p* < 0.05). R software (version 3.2.2; R Foundation, Vienna, Austria) was adopted for statistical analysis [30].

## 3. Results

### 3.1. Systematic Review

**Figure 1:**
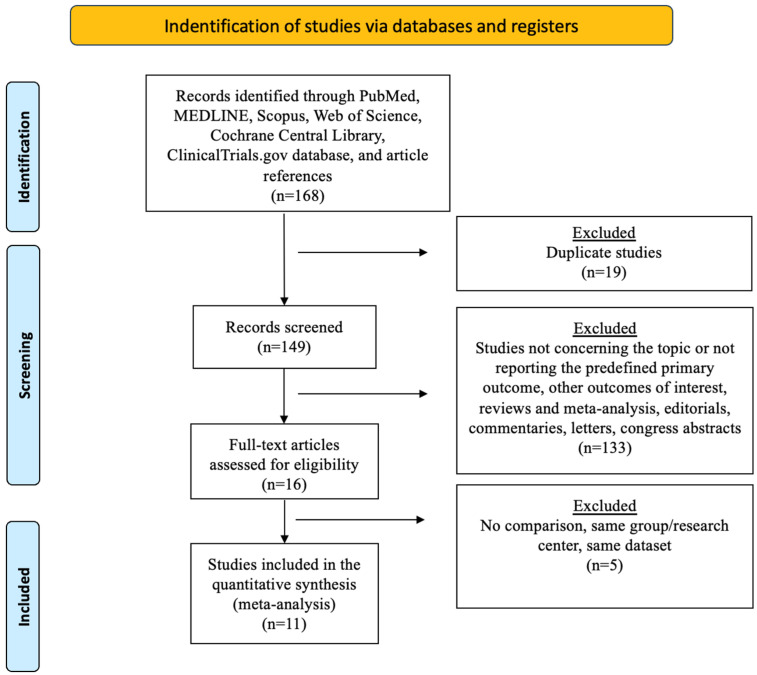
The Preferred Reporting Items for Systematic Reviews and Meta-Analyses (PRISMA) diagram.

The PRISMA flow chart is reported in Figure 1. A total of 168 publications were identified. After duplicates were excluded, 149 titles were screened, and their abstracts were reviewed. After a full text evaluation of 16 articles, 11 studies met the inclusion/exclusion criteria and were used in the quantitative synthesis. All studies were observational, and the specific quality of each study is depicted in Supplementary Table S1.

In total, 1399 patients were included in the study (Table 1) [31,32,33,34,35,36,37,38,39,40,41]. Among them, 576 patients (41.2%) underwent evaluation using ICG during gastric conduit perfusion and anastomosis. The patients’ ages ranged from 22 to 91 years, with 73% being male. Tumour histology was mentioned in eight studies, and the tumour stage based on the AJCC criteria (7th and 8th edition) was outlined in five studies (Stage 0–I: 35.4%, Stage II: 25.1%, Stage III: 31.2%, Stage IV: 8.3%). Details about neoadjuvant chemoradiation therapy were provided in nine studies. The extent of lymphadenectomy (two-field or three-field) was reported in only two studies. The surgical approaches were described in nine studies (Ivor-Lewis: 51.1%, McKeown: 48.9%), as shown in Table 2. Anastomosis types were mentioned in seven studies (circular-stapled in 84.9% of cases). The ICG dosage varied from 2.5 mg to a maximum of 25 mg in seven studies, and the timing of gastric conduit perfusion assessment after ICG injection ranged from 15 to 90 s, as reported in eight studies.

### 3.2. Meta-Analysis: Primary Outcome

**Figure 2:**
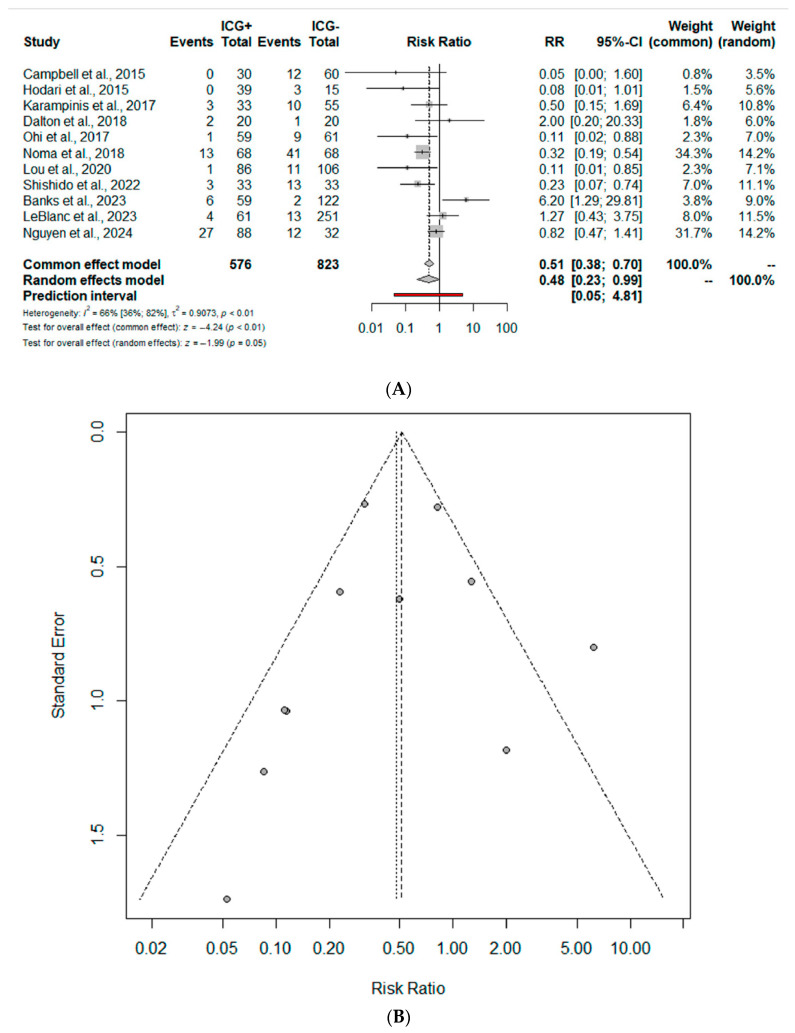
Postoperative anastomotic leak (AL). Forrest plot (**A**) and funnel (**B**) plot. RR: risk ratio; 95% CI: confidence interval [31,32,33,34,35,36,37,38,39,40,41].

AL was reported in 11 studies (1399 patients). The cumulative incidence of AL was 10.4% for the ICG group and 15.4% for nICG. Compared to nICG, ICG esophagectomy was associated with a significantly reduced risk of postoperative AL (RR 0.48; 95% CI 0.23–0.99) (Figure 2A). The prediction lower and upper limits were 0.05 and 4.81, respectively. The heterogeneity was high (I^2^ = 66.0%, 95% CI 36–82%) and τ^2^ = 0.91. The funnel plot (Figure 2B) and the Egger test did not show evidence of publication bias. The sensitivity analysis shows the robustness of these findings in terms of point estimation and heterogeneity.

### 3.3. Meta-Analysis: Secondary Outcomes

The risks of PC (RR 0.83; 95% CI 0.61–1.12; I2 = 28%), OT (SMD −0.47; 95% CI −1.10, 0.15; I2 = 96%), HLOS (SMD −0.16; 95% CI −0.45, 0.13; I2 = 76%), and 90-day mortality (RR 1.70; 95% CI 0.66–4.39; I2 = 30%) were similar between ICG-guided and nICG-guided esophagectomy (Supplementary Figure S1). In the ICG group, a change in management based on perfusion assessments was reported in 10.6% of patients (six studies, 33/327 patients), but only four studies reported the exact incidence of AL in this subgroup (5/33 cases of additional resection, 15.2%) (Supplementary Table S2). Using the GRADE tool, we rated the quality of the evidence of primary outcomes supporting each outcome as moderate, mostly due to limitations in study design (Supplementary Figure S2).

## 4. Discussion

The current systematic review indicates a potential lower risk of post-esophagectomy AL in patients receiving an intraoperative assessment of GC vascularization with intravenous ICG. However, due to allocation bias, variability in AL definition, heterogeneity of surgical approaches and ICG doses, and timing of assessment, caution is recommended to avoid overestimation of ICG effect in reducing AL.

AL after esophagectomy is a recognized complication, with reported incidence rates ranging between 3 and 25% [6,7,42]. Several risk factors have been identified. These include patient-related issues (i.e., age, comorbidities, malnutrition, compromised immune function), anastomotic technique (i.e., cervical vs. intrathoracic, hand-sewn vs. stapled, tension at the anastomotic site, etc.), the experience of the surgical team, the hypoperfusion of the GC, the intraoperative or early postoperative use of vasoconstrictors, epidural catheter utilization, the early initiation of oral intake, prolonged intubation, and neoadjuvant chemo or chemoradiation treatments. Identifying and addressing these risk factors is recommended to possibly reduce the risk of postoperative AL.

Hypoperfusion of the GC has been shown to be a main determinant in the development of postoperative AL. The right gastroepiploic artery is the main vessel supporting GC perfusion after tubulization. Several factors may contribute to GC hypoperfusion such as the surgical technique used for tubulization or a defective intramural vascular network. The perfusion assessment performed during esophagectomy has been a topic of interest for a long time, with several intraoperative techniques proposed to evaluate the GC. However, the results from these methods have been inconsistent, unreproducible, and have shown little correlation with the occurrence of AL [13,14,15,16,43,44]. Since its introduction into clinical practise, ICG has been shown to be an easy, low-cost, and apparently reliable method for the assessment of visceral perfusion [45,46,47,48,49,50,51]. ICG is a tricarbocyanine compound with an excitation spectrum ranging from 700 to 850 nm, and its emission spectrum peaks between 810 and 830 nm. Once injected, its distribution matches the plasma volume, with minimal tissue binding but a high affinity for plasma proteins. The liver clears ICG, which is then excreted by hepatocytes into the biliary system and eventually into the small intestine, where it exits the body through feces without reabsorption. This molecule is taken up by both blood vessels and lymphatic vessels in tissues due to its specific pharmacokinetics [52]. Given these properties, ICG serves as a contrast agent to assess tissue perfusion and aid in visualizing the biliary tract and lymphatic drainage. Since 2012, when Murawa et al. first described its utilization in 15 patients undergoing transhiatal esophagectomy [53], ICG utilization for GC perfusion assessment during esophagectomy has rapidly increased.

In our study, we identified a trend toward a reduced incidence of AL in ICG vs. nICG patients (10.4% vs. 15.8%). Similarly, our quantitative analysis showed a tendency toward reduced postoperative risk for AL in ICG patients (RR = 0.48, 95%CI 0.23–0.99). This is similar to what was described by Zehetner et al., who, in their observational single-centre study, concluded there was a significantly higher risk of AL (45% vs. 2%, *p* < 0.0001) in patients with an anastomosis performed distally to the demarcation of poor perfusion zone [54]. Similarly, Campbell et al. observed a significant reduction in the AL rate for ICG vs. nICG patients (0% vs. 20%, *p* = 0.007) [31]. Additionally, two recent systematic reviews by Ladak et al. and Slooter et al. concluded that, despite the poor quality of data and related heterogeneity, ICG-guided anastomosis appears to be associated with a reduced postoperative incidence of AL [18,54]. Ohi et al. found that the absence of ICG fluorescence in imaging was an independent risk factor for AL in their regression analysis (HR = 9.1; *p* = 0.009). In contrast, Dalton et al. (10% vs. 5%; *p* = NS) and LeBlanc et al. (6.6% vs. 5.2%; *p* = 0.67) did not find a significant difference in the incidence of leaks between an ICG and nICG patient [31,34,38]. Similarly, Casas and colleagues concluded that ICG seems to not be associated with a significantly reduced risk of AL in patients that underwent minimally invasive esophagectomy with intrathoracic anastomosis [19]. In our meta-analysis, despite the point estimation seeming to define a global positive effect in reducing AL for ICG (RR 0.48), the 95% CI upper limit was 0.99. Hence, the exclusive interpretation of the point estimation might be misleading since the upper limit approaches the non-significant threshold (1.00). In addition, the related heterogeneity was moderate (I^2^ = 66%), thus requiring additional attentiveness. Such heterogeneity is presumably attributable to unmeasurable or non-described issues such as technical variations in fashioning the anastomosis (Collard vs. Orringer technique, stapled vs. hand sewn, and linear vs. circular), the use of gastric ischemic preconditioning, omental wrapping, the route of reconstruction (retrosternal vs. posterior mediastinal), baseline comorbidities, smoking status, neoadjuvant treatment, tumour characteristics, tumour-free resection margins (R0), and the surgical approach used (open vs. minimally invasive).

Interestingly, ICG has been advocated for in terms of intraoperative surgical decision making. Van Daele et al. found that 12.4% of ICG patients had a change in surgical management because of sub-optimal perfusion at their anastomotic site [55]. Similarly, Slooter et al. reported a change in surgical management in almost 25% of patients with an additional resection of the poorly perfused tip [56]. In our review, we found that 19.1% of patients (33/173) underwent a change in management after ICG assessment, including an additional GC resection, change in anastomotic site selection, or reconstruction route change (i.e., sternal approach). Interestingly, 15.2% of these patients experienced postoperative AL. This percentage is in in line with what was described by Slooter et al., who reported a 14% AL rate in patients after a change in intraoperative management strategy. This is presumably related to supplementary tension at the anastomosis [55,56,57,58]. Therefore, the seemingly favourable ICG fluorescence imaging does not guarantee an optimal vascular supply, and the possibility of a subsequent AL cannot be excluded, thus suggesting a low negative predictive value.

The lack of objectivity and a specific threshold for adequate perfusion are major limitations related to the utilization of ICG. Specifically, ICG offers qualitative insights, but accurately quantifying tissue vascularization remains a challenge. The light signal’s intensity, serving as a proxy for vascularization, fluctuates depending on the administered ICG dose per bolus (from 1.25 mg to 25 mg), imaging system, and the distance of the laparoscopic optics from the tissue, thus complicating its interpretation. Moreover, factors like GC venous congestion, intraoperative vasoconstrictor utilization, and the route of infusion (peripheral vs. central vessels) can affect the reliability of ICG luminescence. The time to fluorescence has been proposed as an objective adjunctive tool potentially correlated with the risk of postoperative AL. The time from injection to fluorescence observation (i.e., the 60 to 90 s rule), the pattern of flow (i.e., simultaneous vs. delayed), the flow speed cut-off (i.e., 1.76 cm/s), and time to fluorescence–intensity curves have also been proposed, but a robust correlation with AL is lacking. Further, the GC submucosal vascular pattern undergoes dynamic changes within the first 14 days after left gastric and short gastric vessel ischemic preconditioning [11,12]. This is in contrast with the static, instantaneous snapshot provided by ICG imaging, which offers only an initial glimpse of the evolving process. Hence, rather than an assessment of the timing of GC perfusion, future efforts and perspectives should focus on the assessment of the ICG signal’s quantification (SPY-Q technology) and the development of artificial intelligence algorithms to analyze ICG dynamic perfusion, tissue-specific patterns, fluorescent threshold, pharmacokinetics, and clearance 302 time [57,58].

Notably, AL may not solely represent the impact of ICG usage; it is also affected by factors such as surgeon expertise, learning curves, structured training and mentorship programmes, and hospital volume. Both surgical volume and the proficiency of the operating surgeon are crucial for achieving optimal outcomes after esophagectomy. Research indicates that centralizing case loads in high-volume centres significantly lowers mortality rates and may enhance overall results [59]. Notably, during the learning curve, the rate of AL has been shown to decrease from 18% in the initial phase to 4.5% after 119 cases [60]. Considering these factors, it is likely that AL may be influenced by the learning curve effect and surgeon experience, which were not explicitly addressed.

Our study exhibits the typical limitations of a meta-analysis involving observational studies that may be prone to allocation/selection bias, temporal bias, and flaws in study quality. These limitations include the absence of predefined inclusion criteria, variations in surgical approaches, and a lack of globally standardized postoperative management protocols. Additionally, the inconsistency in defining and classifying AL across studies poses a challenge. Furthermore, the influence of surgeon experience and volume on outcomes was not systematically assessed. Finally, the variability in the technical approaches to anastomosis fashioning introduces potential heterogeneity that should be acknowledged.

## 5. Conclusions

ICG assessment of the gastric conduit seems associated with a reduced risk of postoperative AL. Due to diverse study designs, allocation/reporting bias, and variability in AL definition and ICG’s utilization, caution is recommended to avoid overestimation of ICG effect. Future well-designed trials and the use of artificial intelligence and machine learning algorithms to capture complex non-linear relationships may shed light on this topic and improve post-esophagectomy outcomes.

## Figures and Tables

**Table 1:** Demographic and clinical data on patients undergoing esophagectomy with gastric conduit assessment with indocyanine green (ICG) and without indocyanine green (nICG). Ret, retrospective study; Pros, prospective study; PS, Propensity Score; M, male; BMI, body mass index; NT, neoadjuvant therapy; AdK, adenocarcinoma; SCC, squamous carcinoma; NR, not reported.

Author, Year	Study Design	No. Pts	ICG		Gender (M)	BMI (kg/m^2^)	Age (Years)	NT	Histology			Tumour Stage			
									AdK	SCC	Other	1	2	3	4
Campbell et al., 2015 [31]	Ret	90	ICG	30	27	NR	64 (44–81)	3	27	1	2	NR	NR	NR	NR
			nICG	60	47	NR	62 (22–76)	34	48	7	4	NR	NR	NR	NR
Hodari et al., 2015 [32]	Ret	54	ICG	39	NR	NR	NR	NR	NR	NR	NR	NR	NR	NR	NR
			nICG	15	NR	NR	NR	NR	NR	NR	NR	NR	NR	NR	NR
Karampinis et al., 2017 [33]	Ret	88	ICG	33	24	28.8 ± 4.2	65.7 ± 8.5	25	25	7	1	7	10	12	4
			nICG	55	41	25.8 ± 4.3	60.5 ± 8.5	44	NR	NR	NR	17	25	10	3
Dalton et al., 2017 [34]	Pros	40	ICG	20	16	26.4 ± 4.9	61.8 ± 12.8	17	NR	NR	NR	NR	NR	NR	NR
			nICG	20	16	26.3 ± 4.1	66.2 ± 8.0	19	NR	NR	NR	NR	NR	NR	NR
Ohi et al., 2017 [35]	Ret	120	ICG	59	NR	NR	68 (63–74)	NR	NR	NR	NR	NR	NR	NR	NR
			nICG	61	NR	NR	68 (63–74)	NR	NR	NR	NR	NR	NR	NR	NR
Noma et al., 2018 [36]	Ret, PS	136	ICG	68	56	22.1 ± 2.7	65.3 ± 8.1	39	3	62	3	33	17	13	5
			nICG	68	59	22.1 ± 3.4	64.3 ± 7.5	37	2	64	2	31	10	25	2
Luo et al., 2020 [37]	Ret	192	ICG	86	74	22.6 ± 2.9	65.8 ± 6.5	14	2	84	0	28	22	34	2
			nICG	106	97	22.1 ± 3.3	64.9 ± 6.9	12	0	101	5	32	26	42	6
Shishido et al., 2022 [38]	Pros, PS	66	ICG	33	27	21.9 ± 3.3	67 (63–73)	17	2	30	1	13	10	10	0
			nICG	33	30	21.8 ± 3.1	65 (61–73)	17	2	30	1	16	5	12	0
Banks et al., 2023 [39]	Ret	181	ICG	59	46	25.9 ± 4.9	66.9 ± 8.6	52	49	9	1	20	12	20	7
			nICG	122	98	27.3 ± 5.2	64.4 ± 10.2	114	107	14	1	40	30	29	23
LeBlanc et al., 2023 [40]	Pros	312	ICG	61	48	27.5 ± 5.9	70 (51–82)	47	48	10	3	NR	NR	NR	NR
			nICG	251	202	27.3 ± 6.6	67 (30–91)	199	206	33	12	NR	NR	NR	NR
Nguyen et al., 2024 [41]	Ret	120	ICG	88	82	20.3 ± 2.9	62.5 ± 8.1	25	6	81	1	NR	NR	NR	NR
			nICG	32	32	18.9 ± 3.1	63.3 ± 8.1	8	2	30	0	NR	NR	NR	NR

**Table 2:** Operative data on patients undergoing esophagectomy with gastric conduit assessment with indocyanine green (ICG) and without indocyanine green (nICG). IL, Ivor-Lewis procedure; MK, McKeown procedure; O, open thoracotomy; TS, thoracoscopy; R, robot; HS, hand-sewn anastomosis; CS, circular-stapled anastomosis; LS, linear-stapled anastomosis; NR, not reported.

Author, Year	ICG		Procedure		Thoracic Approach			Anastomosis		
			IL	MK	O	TS	R	HS	CS	LS
Campbell et al., 2015 [31]	ICG	30	30	0	2	28	0	0	30	0
	nICG	60	60	0	6	54	0	0	60	0
Hodari et al., 2015 [32]	ICG	39	39	0	0	0	39	0	0	39
	nICG	15	15	0	0	0	15	0	0	15
Karampinis et al., 2017 [33]	ICG	33	25	8	0	33	0	8	25	0
	nICG	55	38	17	0	55	0	17	38	0
Dalton et al., 2017 [34]	ICG	20	20	0	0	20	0	0	20	0
	nICG	20	20	0	0	20	0	0	20	0
Ohi et al., 2017 [35]	ICG	59	NR	NR	NR	NR	NR	NR	NR	NR
	nICG	61	NR	NR	NR	NR	NR	NR	NR	NR
Noma et al., 2018 [36]	ICG	68	0	68	8	60	0	14	49	5
	nICG	68	0	68	21	47	0	14	50	6
Luo et al., 2020 [37]	ICG	86	0	86	NR	NR	NR	0	86	0
	nICG	106	0	106	NR	NR	NR	0	106	0
Shishido et al., 2022 [38]	ICG	33	33	0	0	30	3	0	33	0
	nICG	33	33	0	0	33	0	0	33	0
Banks et al., 2023 [39]	ICG	59	59	0	0	59	0	0	59	0
	nICG	122	122	0	0	122	0	0	122	0
LeBlanc et al., 2023 [40]	ICG	61	NR	NR	0	0	61	NR	NR	NR
	nICG	251	NR	NR	0	0	251	NR	NR	NR
Nguyen et al., 2024 [41]	ICG	88	0	88	0	88	0	NR	NR	NR
	nICG	32	0	32	0	32	0	NR	NR	NR

## Data Availability

The data collected and analyzed during the current review are available from the corresponding author upon reasonable request.

## Supplementary Materials

The following supporting information can be downloaded at https://www.mdpi.com/article/10.3390/jcm13164899/s1, Figure S1. Forrest plot of (A) 90-day mortality, (B) hospital length of stay (HLOS), (C) operative time, and (D) pulmonary complications. RR: risk ratio; 95% CI: confidence interval. Figure S2. GRADE tool for anastomotic leak. Table S1. ROBINS-I. Quality assessment of the included studies (ROBINS-I tool). Each domain is evaluated with one of the following: y “yes”, py “probably yes”, pn “probably no”, or n “no”. The categories of judgement for each study are a low, moderate, serious, and critical risk of bias. Table S2. ICG operative utilization. Timing, time of perfusion assessment after ICG injection; NR, not reported; Mg, milligrams; Sec, seconds.

## Appendix A. Search Strategy

Pubmed

(“Esophagectomy”[Mesh] OR “Esophagectomy”) AND (“Indocyanine Green”[Mesh] OR “Indocyanine Green”)

Scopus

“esophagectomy” AND “esophageal cancer” AND “indocyanine green” AND “fluorescence imaging”

Embase

(‘esophagectomy’ OR ‘esophageal neoplasm’) AND (‘indocyanine green’ OR ICG) AND (‘fluorescence imaging’ OR ‘fluorescence angiography’)

Web of Science

TS=(“esophagectomy” OR “esophageal neoplasm”) AND TS=(“indocyanine green” OR “ICG”) AND TS=(“fluorescence imaging” OR “fluorescence angiography”)

MEDLINE

(“Esophagectomy”[Mesh] OR “Esophagectomy”) AND (“Esophageal Neoplasms”[Mesh] OR “Esophageal Neoplasms”) AND (“Indocyanine Green”[Mesh] OR “Indocyanine Green”) AND (“Fluorescence Imaging”[Mesh] OR “Fluorescence Angiography”)
