# Supplementary material for: Does Indocyanine Green Utilization during Esophagectomy Prevent Anastomotic Leaks? Systematic Review and Meta-Analysis
Source: J Clin Med. 2024 Aug 20;13(16):4899. doi: 10.3390/jcm13164899 (PMC11355508; doi:10.3390/jcm13164899)
Supplement: Supplementary file 1 [file jcm-13-04899-s001.zip › jcm-3115614-supplementary/Appendix 1.pdf]

SEARCH STRATEGY

Pubmed

("Esophagectomy"[Mesh] OR "Esophagectomy") AND ("Indocyanine Green"[Mesh] OR "Indocyanine Green")

Scopus

"esophagectomy" AND "esophageal cancer" AND "indocyanine green" AND "fluorescence imaging"

Embase

('esophagectomy' OR 'esophageal neoplasm') AND ('indocyanine green' OR ICG) AND ('fluorescence imaging' OR 'fluorescence angiography')

Web of Science

TS=("esophagectomy" OR "esophageal neoplasm") AND TS=("indocyanine green" OR "ICG") AND TS=("fluorescence imaging" OR "fluorescence angiography")

MEDLINE

("Esophagectomy"[Mesh] OR "Esophagectomy") AND ("Esophageal Neoplasms"[Mesh] OR "Esophageal Neoplasms") AND ("Indocyanine Green"[Mesh] OR "Indocyanine Green") AND ("Fluorescence Imaging"[Mesh] OR "Fluorescence Angiography")
