# Supplementary material for: Does Indocyanine Green Utilization during Esophagectomy Prevent Anastomotic Leaks? Systematic Review and Meta-Analysis
Source: J Clin Med. 2024 Aug 20;13(16):4899. doi: 10.3390/jcm13164899 (PMC11355508; doi:10.3390/jcm13164899)
Supplement: Supplementary file 1 [file jcm-13-04899-s001.zip › jcm-3115614-supplementary/Suppl Table S1.pdf]

Study	Confonding Bias	Selection Bias	Classification Bias	Intervention Bias	Missing Data Bias	Measurement Bias	Reporting Bias	Bias
Campbell et al., 2015 [31]	py	py	pn	pn	py	pn	pn	Moderate
Hodari et al., 2015 [32]	pn	pn	pn	py	py	pn	py	Serious
Karampinis et al., 2017 [33]	pn	py	pn	py	pn	pn	pn	Moderate
Dalton et al., 2017 [34]	pn	pn	pn	py	pn	pn	pn	Moderate
Ohi et al., 2017 [35]	pn	py	pn	py	pn	pn	pn	Moderate
Noma et al., 2018 [36]	pn	pn	py	py	pn	py	pn	Moderate
Luo et al., 2020 [37]	pn	pn	py	py	pn	py	pn	Moderate
Shishido et al., 2022 [38]	pn	pn	pn	py	pn	pn	pn	Moderate
Banks et al., 2023 [39]	pn	pn	pn	py	pn	pn	pn	Moderate
LeBlanc et al., 2023 [40]	pn	pn	pn	py	pn	pn	pn	Moderate
Nguyen et al., 2024 [41]	pn	pn	pn	py	pn	pn	pn	Moderate

Supplementary Table S1. ROBINS-I. Quality assessment of the included studies (ROBINS-I tool). Each domain is evaluated with one of the following: y “yes”, py “probably yes”, pn “probably no”, and n “no”. The categories of judgement for each study are low, moderate, serious, and critical risk of bias.
