# Supplementary material for: Does Indocyanine Green Utilization during Esophagectomy Prevent Anastomotic Leaks? Systematic Review and Meta-Analysis
Source: J Clin Med. 2024 Aug 20;13(16):4899. doi: 10.3390/jcm13164899 (PMC11355508; doi:10.3390/jcm13164899)
Supplement: Supplementary file 1 [file jcm-13-04899-s001.zip › jcm-3115614-supplementary/Suppl Table S2 def.pdf]

Author, year	System	Perfusion Assessment	Dose (mg)	Timing (sec)	Change in management
**Campbell et al., 2015 [31]**	Spy Elite System	Proximal area of the conduit	5	60	NR
**Hodari et al., 2015 [32]**	Firefly System	NR	NR	NR	NR
**Karampinis et al., 2017 [33]**	PINPOINT System	Time	7.5	NR	6 additional resection, 1 leak
**Dalton et al., 2017 [34]**	PINPOINT System	Time	7.5	60	6 additional resection, 2 leaks
**Ohi et al., 2017 [35]**	PDE	Time	2.5	15-60	9 additional resection, 1 leak
**Noma et al., 2018 [36]**	PDE	Time	NR	30	Anastomosis in perfused zone
**Luo et al., 2020 [37]**	Novadaq	Time	25	60	Anastomosis in perfused zone
**Shishido et al., 2022 [38]**	PDE / Firefly System	Time	10	20	NR
**Banks et al., 2023 [39]**	NR	NR	NR	NR	NR
**LeBlanc et al., 2023 [40]**	NR	Time	NR	48	12 additional resection, 1 leak
**Nguyen et al., 2024 [41]**	NR	Time	5	90	NR

Supplementary Table S2. ICG operative utilization. Timing, time of perfusion assessment after ICG injection; NR, not reported. Mg milligrams. Sec seconds.
