# Supplementary figures and images for: Does Indocyanine Green Utilization during Esophagectomy Prevent Anastomotic Leaks? Systematic Review and Meta-Analysis
Source: J Clin Med. 2024 Aug 20;13(16):4899. doi: 10.3390/jcm13164899 (PMC11355508; doi:10.3390/jcm13164899)

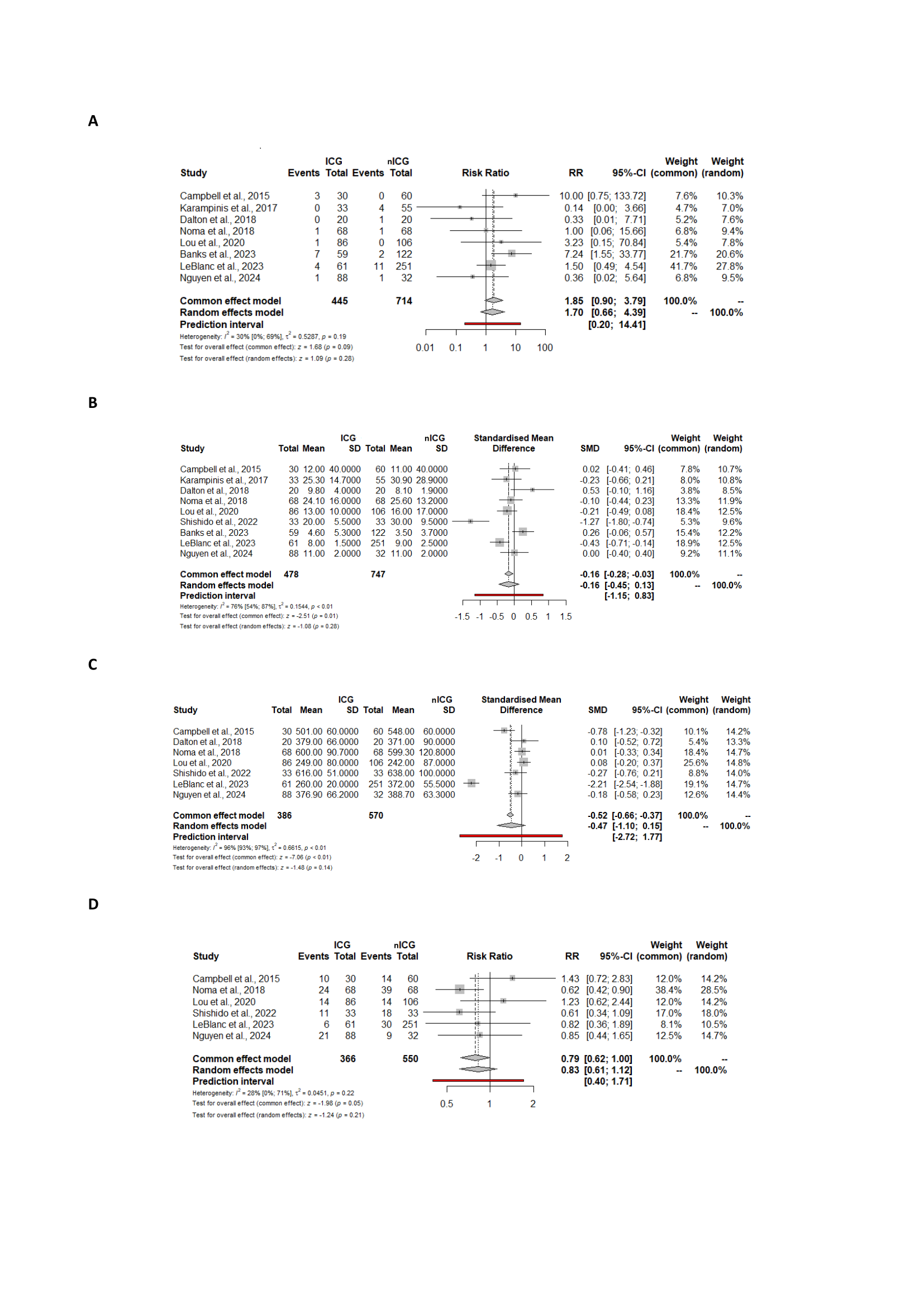

Supplement: Supplementary file 1 [file jcm-13-04899-s001.zip › jcm-3115614-supplementary/Suppl Figure S1.tiff]

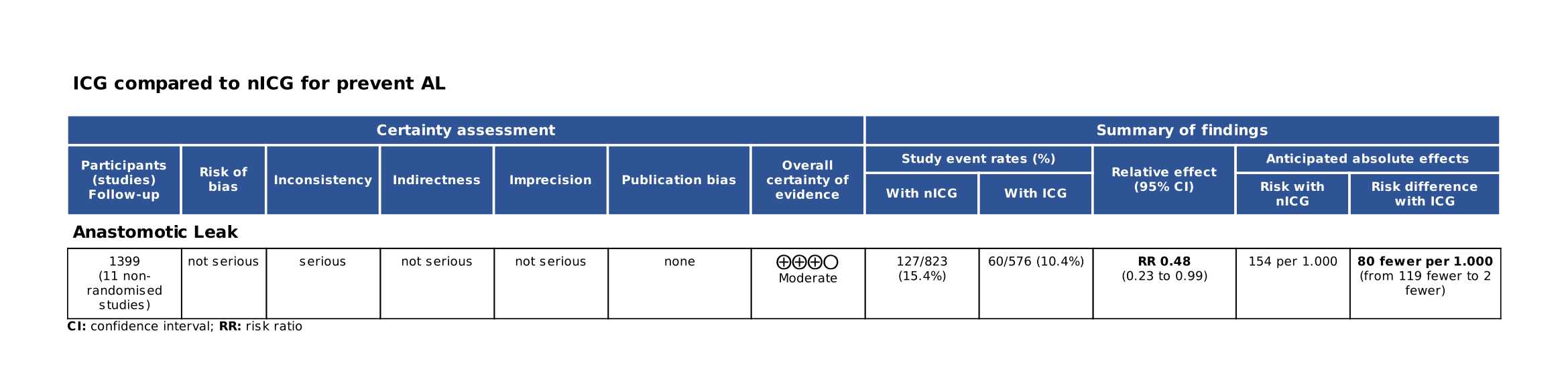

Supplement: Supplementary file 1 [file jcm-13-04899-s001.zip › jcm-3115614-supplementary/Suppl Figure S2.tiff]
